# Development of an MRI-Compatible Nasal Drug Delivery Method for Probing Nicotine Addiction Dynamics

**DOI:** 10.3390/pharmaceutics13122069

**Published:** 2021-12-03

**Authors:** Rajat Kumar, Lilianne R. Mujica-Parodi, Michael Wenke, Anar Amgalan, Andrew Lithen, Sindhuja T. Govindarajan, Rany Makaryus, Helene Benveniste, Helmut H. Strey

**Affiliations:** 1 Department of Biomedical Engineering, Stony Brook University School of Medicine, Stony Brook, NY 11794, USA; rajatk489@gmail.com (R.K.); lilianne.strey@stonybrook.edu (L.R.M.-P.); MWENKE@mgh.harvard.edu (M.W.); anar.amgalan@alumni.stonybrook.edu (A.A.); Andrew.lithen@alumni.stonybrook.edu (A.L.); Sindhuja.TirumalaiGovinddarajan@PennMedicine.upenn.edu (S.T.G.); 2 Department of Radiology, Athinoula A. Martinos Center for Biomedical Imaging, Massachusetts General Hospital, Charlestown, MA 02129, USA; 3 Department of Anesthesiology, Stony Brook University School of Medicine, Stony Brook, NY 11794, USA; Rany.Makaryus@StonyBrookMedicine.edu (R.M.); helene.benveniste@yale.edu (H.B.)

**Keywords:** nasal drug delivery device, nicotine addition, brain circuit

## Abstract

Substance abuse is a fundamentally dynamic disease, characterized by repeated oscillation between craving, drug self-administration, reward, and satiety. To model nicotine addiction as a control system, a magnetic resonance imaging (MRI)-compatible nicotine delivery system is needed to elicit cyclical cravings. Using a concentric nebulizer, inserted into one nostril, we delivered each dose equivalent to a single cigarette puff by a syringe pump. A control mechanism permits dual modes: one delivers puffs on a fixed interval programmed by researchers; with the other, subjects press a button to self-administer each nicotine dose. We tested the viability of this delivery method for studying the brain’s response to nicotine addiction in three steps. First, we established the pharmacokinetics of nicotine delivery, using a dosing scheme designed to gradually achieve saturation. Second, we lengthened the time between microdoses to elicit craving cycles, using both fixed-interval and subject-driven behavior. Finally, we demonstrate a potential application of our device by showing that a fixed-interval protocol can reliably identify neuromodulatory targets for pharmacotherapy or brain stimulation. Our MRI-compatible nasal delivery method enables the measurement of neural circuit responses to drug doses on a single-subject level, allowing the development of data-driven predictive models to quantify individual dysregulations of the reward control circuit causing addiction.

## 1. Introduction

Nicotine is the most common drug of abuse in the United States [1], with addiction strength comparable to cocaine, heroin, and alcohol [2,3]. It is the primary addictive component of tobacco, and its use markedly increases risk for cancer, heart disease, asthma, miscarriage, and infant mortality [2]. Excitatory vs. inhibitory control plays a key role in addictive behavior; for example, decreasing glutamate or increasing gamma aminobutyric acid (GABA) transmission blunts nicotine craving in rats [4,5]. Importantly, however, substance abuse is a fundamentally *dynamic* disease, characterized by repeated oscillation between craving, drug self-administration, reward, and satiety. While there is a general appreciation for the heuristic value of separating out these stages [6], current clinical research has primarily focused on identifying nodes and causal connections within the meso-circuit of interest, but has yet to take the next step in treating these nodes and connections as a self-interacting dynamical system evolving over time [7,8]. The value of a dynamical systems approach is the potential for predicting trajectories for addiction as well as recovery. These trajectories are likely to be nonlinear (e.g., involving thresholds, saturation, and self-reinforcement) and individual-specific.

As a first step towards this approach, we identified several requirements for nicotine delivery. First, the delivery method should be able to mimic the pharmacokinetics of the addictive substance, as there is significant evidence that absorption speed affects cravings [9]. Second, delivery should be capable of eliciting multiple cycles that transition between craving, resistance, breakdown of resistance, and satiety, as a prerequisite to data-driven computational modeling of feedback within the control circuit. Third, craving cycles—and associated behavioral self-administration—should be triggered solely on drug pharmacokinetics and their dynamic interactions with the brain. This has the advantage of decoupling chemical effects from cigarette-specific motor, olfactory, and visual cues, each of which may trigger its own responses in the brain. Fourth, to permit investigation of the relationship between drug-seeking behavior and its underlying neurobiology, it should allow subjects to self-administer nicotine. Fifth, the device should be MRI-compatible, as functional MRI (fMRI) is currently the only non-invasive neuroimaging tool with subcortical-cortical coverage of the entire prefrontal-limbic reward circuit. While neuroimaging has historically relied on heavily fitted and filtered amplitude and correlational statistics, recent technological developments in ultra-high-field (7T) fMRI and simultaneous multi-slice pulse sequences, provide sufficient signal/noise to permit single subject, non-trial-averaged, and sub-second time-resolution time-courses that retain much greater dynamic information [10,11].

To administer nicotine within a controlled environment, researchers have employed one of five strategies. These are: (1) *smoking* (either directly, or through an apparatus that ensures controlled delivery), (2) *intravenous* (either as a bolus injection or continuously by drip), (3) *transdermal* (using a nicotine patch), (4) *absorption through the lungs* (nicotine solution nebulized in mouth), or (5) *absorption through the mucus membranes* (nicotine solution sprayed in nose/throat) [12].

Several of those strategies introduce additional practical problems and scientific confounds. Although other groups have designed MRI-compatible smoke delivery devices for use in 3T scanners [13], this method can raise environmental safety concerns regarding second-hand smoke inhalation and lingering odor. Moreover, tobacco is a natural substance with non-standardized composition, and thus prevents precise control of dosage. Nicotine patches are noninvasive and minimize discomfort from nicotine. However, their extended release of nicotine also renders their pharmacokinetics different from smoking, and do not elicit cravings. Inhaling nebulized nicotine by mouth would intuitively seem to approximate the act of smoking, but again the pharmacokinetics do not translate well to cigarettes. The delivery efficiency of nebulizers also depends strongly on how deeply liquid droplets can penetrate the lung, which itself is determined by size and aerosol flow rate [14]. Delivery by e-cigarettes uses nicotine concentrations in their solutions that are much higher than those of cigarettes because of the relative inefficiency of the lung to absorb liquid droplets. Heating can miniaturize the droplets; however, heating elements are electric, and therefore are not MRI compatible. Intravenous bolus has pharmacokinetics closer to those of smoking [15] but the technique is invasive and may be harder to justify to an institutional review board (IRB).

In recent years, nasal delivery has emerged as a promising strategy to deliver drugs into the brain [16,17,18,19,20,21,22,23]. The most prominent nasal delivery route is by absorption into the bloodstream through the highly vascularized nasal cavity through venous drainage. Nasal delivery, therefore, has similar kinetic characteristics as intravenous delivery. In addition, several other nasal-to-brain drug delivery routes that circumvent the blood–brain barrier have been reported [16,17,24]. Nasal nicotine delivery is well characterized both in terms of kinetics and efficiency of delivery [25] with several commercial nicotine sprays being on the market. For example, the commercial nicotine replacement medication Nicotrol NS (Pfizer, New York, NY, USA) delivered through the nose, shows significantly greater nicotine craving relief than the same Nicotrol inhaler inhaled through the mouth [12]. On the other hand, nicotine nasal sprays are reported to cause nasal irritation, watery eyes and coughing. Such adverse effects are especially bad for MRI since such irritations will lead to head motion. However, we found that by spraying Lidocaine into the nasal cavity before the experiment those irritations could be completely suppressed for the duration of the experiment.

To exploit the advantages of nasal nicotine delivery, we designed a delivery device to operate as a concentric nebulizer, inserted into one nostril. We delivered each dose—each equivalent to a single cigarette puff—using a syringe pump and nebulized the dose using pressurized medical air. A control mechanism permits dual modes: one delivers puffs on a fixed interval programmed by researchers; with the other, subjects press a button to self-administer each nicotine dose. Subjects were therefore able to intuitively “smoke” the equivalent of a cigarette, one “puff” at a time. We dosed each “puff” such that one cigarette would be equal, in nicotine content, to 10 puffs. We then tested the viability of this delivery method for studying the brain’s response to nicotine addiction in three steps. First, we established the pharmacokinetics of nicotine delivery, using a dosing scheme designed to gradually achieve saturation, as with a cigarette. Second, we lengthened time between microdoses to elicit craving cycles, using both fixed-interval and subject-driven behavior. Finally, we demonstrate a potential application of our device by showing that a fixed-interval protocol can reliably identify neuromodulatory targets for pharmacotherapy or brain stimulation.

## 2. Materials and Methods

### 2.1. Delivery Apparatus Design

**Figure 1:**
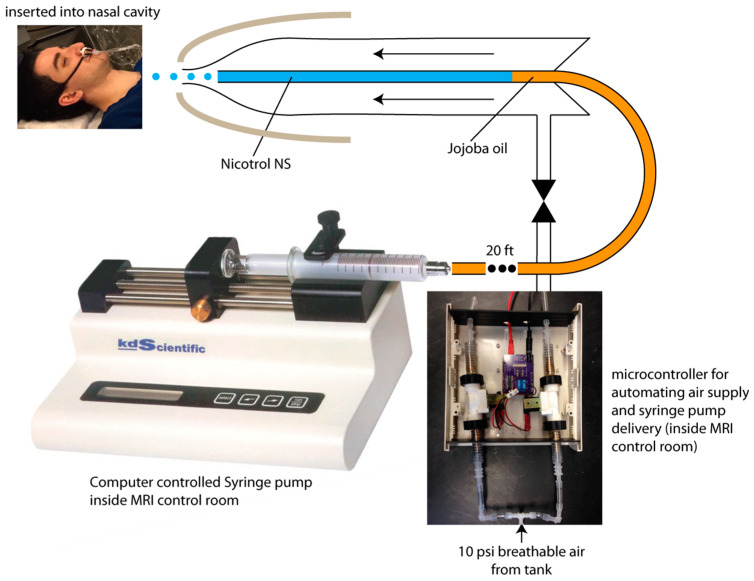
Schematic of the MRI-compatible intranasal nicotine delivery apparatus.

The nicotine delivery apparatus uses a glass concentric mass spectroscopy nebulizer (Model TR-30-A1, Meinhard, Colden, CO, USA) to deliver Nicotrol nasal spray (NS) into the subject’s nasal cavity. This setup can be used to deliver any water-soluble drug intranasally in an MRI environment (Figure 1). A wide nostril guard (Aptar Pharma, Pennsauken, NJ, USA) and drawstring strap were used to orient the nebulizer straight in the nose and to ensure the nebulizer does not penetrate too deeply. The nebulizer’s inner capillary contained the Nicotrol NS, which was driven out of the capillary as a mist when medical air is driven through the outer chamber. Medical air pressure was held constant at 15PSI with a regulator (VWR Breathair, Randor, PA, USA). This was found to be the optimal air pressure for producing a fine mist of Nicotrol NS that deposits deep enough in the turbinate for rapid absorption while maximizing subject comfort [26]. At the flow conditions used the average droplet size exiting the nebulizer are expected to be in the range of 20–30 µm [27]. Given that the nebulizer produces a wide distribution of droplets sizes and since we ask the subjects to breath in through the nose and breath out through the mouth, we cannot completely rule out some tracheobronchial or pulmonary delivery of nicotine solution. The medical air flowed through a length of Tygon Non-DEHP Food and Beverage Tubing (S3-B-44-3, 3/16”x5/16”, Cole Parmer, Vernon Hills, IL, USA) and then through an air valve (Clippard MME-32QES, Airoyal, Maplewood, NJ, USA), which only allowed air to reach the nebulizer during puff delivery. A second air valve was used to quickly depressurize the tubing at the termination of each puff. The Nicotrol NS was dispensed in 10µL puffs, each lasting three seconds, using an automated syringe pump (KDS100, KD Scientific, Holliston, MA, USA). This syringe pump compressed a Hamilton glass syringe (Fischer Scientific, Waltham, MA, USA), which was connected to a length of capillary tubing (F2-15, 0.8 mm inner diameter, Meinhard, Colden, CO, USA) and a three-way valve (PEEK 3-Port Flow, V100T, Idex Health Science, Bristol, CT, USA) before feeding into the nebulizer. Nicotrol NS sat in the length of tubing adjacent to the nebulizer, while the remainder of the tubing feeding back to the syringe pump was filled with cosmetic jojoba oil. This oil was used because it is incompressible and immiscible with the Nicotrol NS, allowing for accurate delivery of a specific volume of Nicotrol.

An Arduino Uno microcontroller coordinated the release of puffs. The experimental task, which was programmed in MATLAB (R2018a; Mathworks, Natick, MA, USA), sent a signal to this microcontroller every time a puff was to be delivered. At this point, the Arduino unit triggered the following events. First, the microcontroller opened the air valve for 16 s, allowing air pressure to build up in the nebulizer. A countdown was shown to the subject indicating when nicotine would be delivered. At the end of the countdown, the microcontroller then signaled the syringe pump to compress 10 µL over 3 s, pushing 10 µL of Nicotrol NS out of the nebulizer as a mist. The air valve remained open for an additional 16 s after the end of the previous countdown to ensure all Nicotrol at the nebulizer tip was delivered. Finally, the main air valve was closed, and the exhaust valve opened to quickly depressurize the tubes carrying air. The task screen reverted to the cravings indicator.

### 2.2. Preparing the Apparatus for Experimentation

Immediately prior to use on any subject, clean and sterilized capillary tubing was filled with jojoba oil and Nicotrol while carefully avoiding to trap air in the path to ensure the accuracy of Nicotrol NS delivery. The length of capillary tubing leading to the Hamilton syringe was filled with jojoba oil by submerging the capillary end connecting to the three-way valve in oil; we then used the Hamilton syringe to draw oil to fill the capillary tube. Leaving an oil bead at the end of the capillary tube proximal to the syringe pump, the Hamilton syringe was disconnected, fully compressed, reconnected, and pulled back to fill with oil while excluding any air. A volume of 300 µL Nicotrol NS was drawn in an Eppendorf tube and spun down using a microcentrifuge to remove any air bubbles. This volume contained 3 mg nicotine, the amount of nicotine present in three typical cigarettes (the potential for nicotine overdose was thus eliminated since this was the maximum dose that could be delivered). A dyed jojoba oil solution was prepared by mixing 1 mL of oil with a few drops of food coloring (Wilton Candy Colors) and homogenizing with a vortex mixer or sonication. The short capillary end of the three-way valve was submerged in the Nicotrol NS. A 1 mL draw syringe was connected to the long capillary end of the three-way valve and pulled back until all the Nicotrol NS was drawn into the capillary tube. Leaving a Nicotrol bead at the short capillary end, this end was submerged in the dyed jojoba. The draw syringe was slowly drawn up until the long capillary end was filled. Slow filling was needed to reduce viscous fingering at the interface of the Nicotrol NS and dyed oil. The syringe pump was compressed to produce a bead at the end of the connected capillary tube. This capillary was then screwed into the three-way valve with the valve to the syringe pump closed off. The three-way valve was rotated 90 degrees to close off the capillary leading to the nebulizer and connecting the syringe pump capillary to the short capillary end. This releases the built-up pressure from screwing in the syringe pump capillary. The syringe pump was then compressed until undyed oil was visible in the short capillary tube, indicating no air is present in the three-way valve. The draw syringe was replaced with the nebulizer. The three-way valve was rotated 180 degrees to close off the short capillary end and make a continuous path from the syringe pump to the nebulizer. The syringe pump was then compressed until Nicotrol NS filled the inner capillary of the nebulizer. The nebulizer, nose cone, and strap were sanitized and assembled. The nebulizer was then positioned in the subject’s nostril. At the termination of each experiment, the three-way valve, nebulizer, associated capillary tubes, and nose cone were disinfected by passing through with 20 mL soap water, and then 10 mL Sporgon disinfectant (Decon Laboratories, King of Prussia, PA, USA). The assembly was then submerged in a Sporgon bath for 3 h, and then rinsed with 20 mL deionized water. The assembly tubing was then dried using pressurized medical air.

### 2.3. Subjects

To establish pharmacokinetics of our delivery mechanism, we tested the device with dynamic assessment of cravings and two blood sampling regimes in participants at Stony Brook University School of Medicine (*Studies A, B*). To test whether the delivery method could trigger craving cycles that, in turn, were linked to drug-administration, we repeated the experiment with only dynamic cravings assessment at the Massachusetts General Hospital A.A. Martinos Center for Biomedical Imaging. This was initially done using lengthened inter-trial intervals (*Study C*), and then, using *ad libitum* self-administration (*Study D*). Finally, to confirm that the delivery method activated the reward circuit, we scanned individuals’ response to nicotine puffs using ultra-high-field/ultra-fast fMRI at the Massachusetts General Hospital A.A. Martinos Center for Biomedical Imaging (*Study E*). Characterization of subjects for each of the five studies is shown in Table 1.

For all studies, we recruited subjects who were otherwise healthy daily smokers with moderate to severe nicotine dependency, and therefore who would be likely to show strong cravings following the 12 h abstinence period preceding each session. Nicotine dependency was measured on a scale of 0–10 using the Fagerstrom Test for Nicotine Dependence (FTND) [28]. For all studies, we included only subjects with FTND scores ≥ 6, ages 21–55 with a BMI of 18.5–35. Abstinence prior to testing was empirically confirmed by measuring exhaled carbon monoxide levels using a Micro+ Basic Smokerlyzer (Covita, Santa Barbara, CA, USA), and further confirmed via blood testing. Subjects with measured baseline carbon monoxide levels > 10 ppm were excluded prior to testing; any remaining subjects with baseline nicotine levels > 10 ng/mL were excluded prior to analysis. Additional exclusion criteria included nasal congestion, sinusitis, use of nicotine cessation therapy medications, history of asthma, cardiovascular, or peripheral vascular disease, history of neurological disease, or the use of psychotropic medications. These criteria would influence the absorption of nicotine through the nasal mucosa, its distribution throughout and dissipation from the blood stream, and its effect on specific brain regions [29]. Additional exclusion criteria concerning MR safety included electrical implants, ferromagnetic implants, claustrophobia, and pregnancy. Exclusion due to pregnancy was determined using a urine pregnancy test (Detector hcg, Immunostics Inc, Eatontown, NJ, USA). Studies were approved by the Institutional Review Boards of Stony Brook University and Massachusetts General Hospital. All subjects provided written informed consent.

### 2.4. Task Design

For all studies, subjects were nicotine addicted and had abstained for at least 12 h (validation methods described above). Immediately prior to the start of the testing session, we administered 4% Lidocaine HCl (Roxane Laboratories, Columbus, OH, USA) in the subject’s nasal cavity to alleviate the irritation from Nicotrol delivery. Lidocaine was delivered using an intranasal mucosal atomization device (Mountainside Medical Equipment Incorporated, Marcy, NY, USA). Behavioral trials used a 1 mL Lidocaine dose, while MRI trials used a 3 mL dose due to the increased length of the testing session. The experimental task was developed using MATLAB and Psychtoolbox-3. Behavioral task sessions ran for 60 min, divided into five minutes of baseline measurements, 40 min of the main task with puff delivery, and 15 min of end of study observation.

#### 2.4.1. Pharmacokinetic Studies

*Study A* provided estimation of the entire time-course, while *Study B* was designed to capture the faster-changing dynamics during uptake. For the first, we presented 20 nicotine puffs, one every two minutes, and took seven blood samples: at baseline, after every four puffs (blood sampling every eight minutes), and 15 min post-administration of the last puff. For the second, we maintained the same sampling at baseline and 15 min post-administration of the last puff, but this time sampled after every two puffs (blood sampling every four minutes).

#### 2.4.2. Cravings Studies

To test whether the delivery method could trigger craving cycles that, in turn, were linked to drug-administration, we repeated the experiment with only dynamic cravings assessment. This was initially done using lengthened inter-trial intervals [30] (*Study C*), and then, using *ad libitum* self-administration (*Study D*). For *Study C* we used the same 10-puff protocol, but delivered puffs every four minutes, with blood sampling only at baseline to confirm abstinence. We used longer ITIs to ensure that the reward circuit had sufficient time to resolve following each puff, thereby eliciting serial craving cycles without ever achieving full satiety. For *Study D*, one puff was delivered at the beginning of the scan, and subjects were instructed to only request additional puffs when they could no longer resist their cravings. Subjects were not permitted to request a puff more frequently than once every two minutes; thus, subjects could request a maximum of 20 puffs.

#### 2.4.3. Neuroimaging

Finally, to show a potential application of our delivery method in probing nicotine addiction, we infer the targets for neuromodulation via measured neurobiological response to nicotine puffs using ultra-high-field/ultra-fast fMRI at the Massachusetts General Hospital A.A. Martinos Center for Biomedical Imaging (*Study E*). We used the 10-puff protocol used in *Study C*, delivering puffs every four minutes, and blood sampling only at baseline to confirm abstinence.

### 2.5. Dynamic Cravings Assessment

For *Studies A–E*, throughout each 60 min session, subjects indicated their relative cravings intensity on a dynamic Likert scale from “0” to “100,” with “0” corresponding to “no cravings” and “100” corresponding to “strongest cravings imaginable.” Likert scales are commonly used in assessing craving in the context of addiction [31,32]. The craving scale was represented by a continuously updated line (TR = 1 s), showing the subject’s cravings over the past five minutes. Subjects used a button box to adjust cravings-ratings throughout each session.

### 2.6. Continuous Blood Sampling

For *Studies A, B*, arterialized venous blood samples were collected throughout each 60 min behavioral session as a less invasive alternative to arterial sampling [33]. An intravenous line was placed in the subject’s non-dominant hand, and the hand was placed in a warming box at 50 °C as described in [33]. Each blood draw collected 4 mL of blood, which was immediately transferred to a Vacuette Clotting Tube (Greiner Bio-One, Monroe, NC, USA) for serum separation. Prior to the start of the 60 min session, the first blood draw was drawn to determine baseline nicotine and nicotine metabolite levels. Throughout the 40 min puff delivery period, five additional blood samples were collected. In the 20-puff protocol, blood was drawn one minute after Puffs 4, 8, 12, 16, and 20. In both 10-puff protocols, blood was drawn one minute after Puffs 2, 4, 6, 8, and 10. One final blood sample was collected at the end of the 60 min session for all behavioral protocols. During MRI trials, no heating box was used and no IV was placed, and only the first baseline blood draw was collected. Serum was separated from these samples and analyzed by ARUP Laboratories (Salt Lake City, UT, USA) for *nicotine*, *cotinine*, and *trans-3’-hydroxycotinine* levels.

### 2.7. Neuroimaging

#### 2.7.1. Acquisition Parameters

Our use of ultra-high field strength, Siemens Magnetom 7 Tesla scanner with 32-channel head coil, combined with EPI acquisition parameters (repetition time—TR = 802 ms, echo time—TE = 30 ms, 85 slices) optimized by a dynamic phantom for dynamic fidelity, were chosen to maximize single-subject-level detection sensitivity of prefrontal-limbic and reward circuits [10,11]. Structural scans, for spatial co-registration, were acquired as multi-echo magnetization prepared—rapid gradient echo (MPRAGE) with 1 mm isotropic voxel size at four echoes with TE1, TE2, TE3, TE4 = 1.61, 3.47, 5.33, 7.19 ms, TR = 2530 ms, flip angle = 7 degrees, slice gap = 0.5 mm and generalized autocalibrating partial parallel acquisition (GRAPPA) acceleration = 2. B0 field map images calculated using phase differences between gradient echo images at TE = 4.60 and 5.62 ms, were also acquired (TR = 723 ms, flip angle = 36°, voxel size = 1.7 × 1.7 × 1.5 mm, 89 slices) for echo-planar imaging [34] distortion correction arising due to magnetic field inhomogeneity.

#### 2.7.2. Preprocessing

Spatial preprocessing was performed in Statistical Parametric Mapping (SPM12). Functional images were corrected for motion (rigid realignment, 6 degrees of freedom) and a mean functional image was calculated for each subject. These mean functional images were co-registered to high-resolution structural images followed by segmentation to generate gray matter, white matter and deformation field images. The realigned (field map corrected) functional images were normalized to Montreal Neurological Institute (MNI) EPI template with affine registration followed by a non-linear transformation (between average fMRI and EPI template). Finally, the images were smoothed with a Gaussian kernel of 4 mm at full width at half maximum. Correction for static field inhomogeneity was performed after the realignment step using a field map-based EPI unwarping tool built in FSL [35]. Nicotine is known to cause vasoconstriction and alters heart rate/blood pressure as well as respiration rate [36]. The vascular effects on blood-oxygen-level-dependent [37] signal can be reduced by using nuisance regressors derived from cerebrospinal fluid (CSF) and white matter [38,39] that are unlikely to show neural activity induced T_2_ * (time constant for observed decay of transverse magnetization) changes. We used the CompCor method [40], implemented using CONN toolbox [41], to account for the effect of physiological noise on the BOLD signal. CompCor regresses out the confounding effects of multiple empirically estimated noise sources calculated from variability in BOLD time series of cerebrospinal fluid and white matter (principal component analysis). Five components of white matter, five components of the cerebrospinal fluid and six motion parameters, along with despiking and quadratic detrending, were used for denoising.

#### 2.7.3. Finding Neuromodulatory Targets

Neural systems are fundamentally organized as large-scale networks composed of computational and modulatory units existing at multiple spatial scales. Network controllability [42] provides a mechanistic ground for impinging region/node-level perturbations to drive a dysregulated brain state to the desired state of better health. Clinically, applying perturbations would mean manipulating distributed neurotransmitter systems via pharmacological intervention or brain stimulation. As a single region generally interacts with multiple other regions within a network, targeting individual regions using pharmacotherapy/brain stimulation affects the global dynamics of the network. Given that the topology of functional networks provides an insight on how external inputs can drive the brain to different states [43], it is quintessential to identify regions/nodes that renders a network controllable (ability to drive a network from an initial state to a desired final state in finite time). Psychopathology induces hysteresis [44] wherein once a system is driven into a diseased state, it tends to remain in that state unless external intervention triggers a change back to a healthy state. If dynamic graphs constructed for both the abstinence and the satiety condition come from a diseased state (addicted subjects), the brain-connectivity graphs (brain connectivity of the diseased state) encoded by abstinence and satiety should be controllable from a small set of common driver nodes [44] across addicted subjects. Obtaining such driver nodes will allow for neuromodulation for driving the brain to a healthy state.

The ten puff (fixed interval) protocol, delivering a puff every four minutes as in *study C*, was used during neuroimaging. We extracted a time series for each subject for previously established regions/nodes (23 regions) associated with the addiction [45]. Each region’s time series was split into two conditions for every subject, namely, abstinence and satiety. The first condition, abstinence, consisted of 380 TRs (304.76 s) acquired before administering nicotine puffs in the baseline measurement condition. The fMRI time series for the first condition was then split into 10 windows of 38 TRs (30.47 s) each for each region. The second condition, satiety, consisted of a total of 380 TRs, split across 10 windows of 38 TRs (30.47 s), with each window representing a time series starting 30.47 s after each nicotine puff. We then used the following procedure on windows for both the abstinence and satiety condition. First, we formed 10 weighted connectivity matrices (23 × 23) by calculating pairwise Pearson correlation among regions within a window, followed by calculation of the nodal connectivity strength of each region for each window (10 × 23). We then calculated the Pearson correlation of nodal connectivity strength between any pair of windows across regions, yielding a 10 × 10 matrix. We reordered the 10 × 10 matrix using hierarchical clustering [46] to compute brain-connectivity states. Lastly, we binarized each brain-connectivity state (threshold = 0.65), followed by computation of the minimum dominating set [47] for each binarized brain-connectivity state to find the dominant driver nodes/neuromodulation targets. Driver nodes, which are common across all brain-connectivity states, were used for finding average number of slave/controlled nodes across all brain-connectivity states and the top two nodes were picked as neuromodulation target sites.

## 3. Results

### 3.1. Our Nasal Nicotine Delivery Method Showed Blood Levels Consistent with Values from the Literature

**Figure 2:**
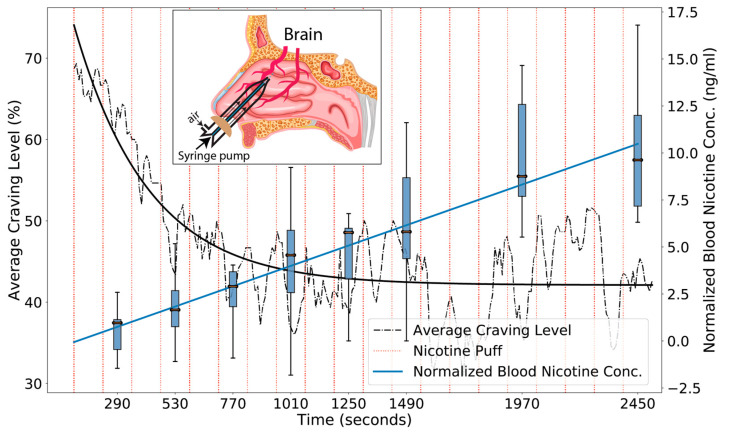
Nasal nicotine administration every 2 min linearly increases nicotine concentration and exponentially decreases subjective cravings. On average each spray of 10 µL delivers 0.6 ng/mL into the blood stream, with a delivery efficiency of 3.4% (*Studies A, B*).

As can been seen from Figure 2, the normalized arterialized blood nicotine levels reached between 7.5 and 12 ng/mL after ten puffs or 1 mg of nicotine (in Nicotrol NS). This is about half of the average arterial blood nicotine concentration for smoking as reported in [15]. Our values are consistent with venous blood nicotine values reported in [25] for nasal delivery of similar dosing (between 5 and 20 ng/mL). Part of the reason for this discrepancy could be related to the difference of arterialized blood and arterial/venous blood. There is some evidence that arterialized blood lies somewhere in between arterial and venous blood [48]. In addition, we draw blood one minute after every two/four puffs and would miss any nicotine peaks in arterial blood.

### 3.2. Our Nicotine Delivery Method Showed Corresponding Effects on Cravings

**Figure 3:**
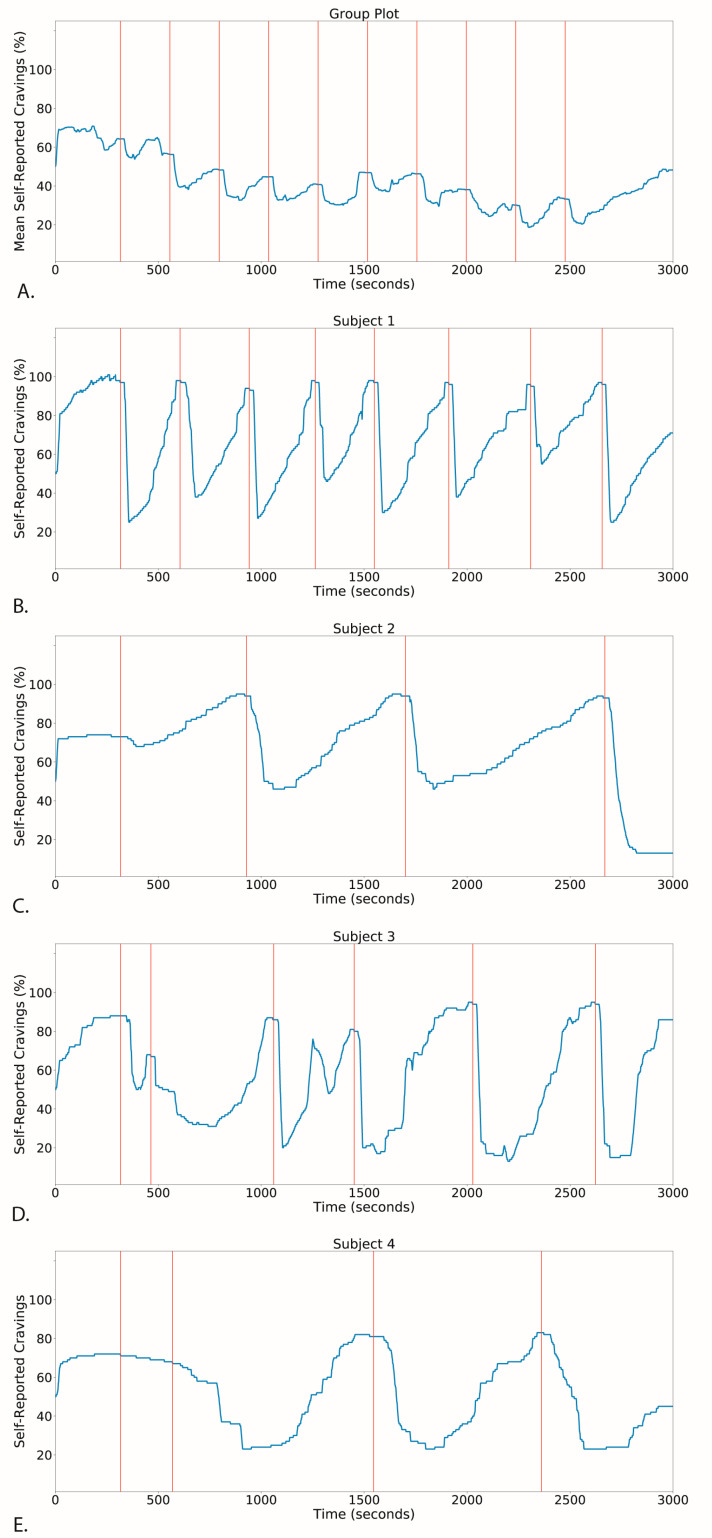
*Craving Cycles.* Nasal nicotine microdosing elicits craving cycles for fixed inter-trial intervals (4 min) (**A**, Craving averaged over all participants in *Study C*) and predicts individual dynamics of self-administration (**B**–**E**, *Study D*).

As shown in Figure 2, with more rapid (ITI = 2 m) puff administration, cravings decreased exponentially with each puff. With an exponential fit, A + B × exp(−t/τ): A = 4.2 +/− 0.07, B = 3.2 +/− 0.3, and τ = 310 s +/− 45 s. Since the nicotine delivery was linear in time, and cravings reduced exponentially, the relationship between cravings and nicotine was also exponential. Each puff delivered 0.6 ng/mL into the blood, and the decay time was 310 s, or 2.6 puffs. In terms of nicotine, 1.55 ng/mL was the exponential decay concentration. Therefore, our observed relationship with this delivery method was: cravings = A + B × exp(−*nicotine concentration*/1.55 ng/mL). As shown in Figure 3, by doubling duration between puffs (ITI = 4 m), we were able to achieve oscillating transitions between cravings and satiety that, for our *ad libitum* design, corresponded with behavioral self-administration of nicotine.

### 3.3. Anterior Cingulate Cortex and Nucleus Accumbens Are the Two Most Dominant Driver Nodes across Subjects and Hence Are Suitable Neuromodulatory Targets in Treatment for Nicotine Addiction

Our results provide a significant overlap with the emerging literature on neuromodulatory targets implicating the anterior cingulate cortex (ACC) [49] and the nucleus accumbens (NAcb) [50,51,52] for treatment of substance use disorders. Specifically, we found the driver nodes of brain-connectivity states, under two different conditions of abstinence and satiety, using the minimum dominating set analysis. Driver nodes form suitable neuromodulation targets because each driver node can control the regions it is connected to and thus, neuromodulation at driver nodes can alter the underlying neuronal circuits at a synaptic level. As show in Table 2, ACC consistently appears to be the most dominant driver node (averaged across brain states) across subjects, with controlling 75.36% of the regions for subject 2. Nucleus accumbens is the second most dominant node with controlling 42.39% of the regions for subject 1. Additionally, we consistently observed the *left-hemisphere* NAcb to be the driver node across all subjects—suggesting an asymmetric role of the left and the right NAcb in addiction. Lastly, we found that ACC and NAcb are the dominant driver nodes for both the brain-connectivity states in abstinence and the satiety, suggesting pathological hysteresis wherein the diseased state [1] influences the brain dynamics, and hence the brain-connectivity states, more than the temporary change in external influence (administering nicotine puffs).

## 4. Discussion and Conclusions

Our results successfully established the pharmacokinetics of nicotine delivery, using a dosing scheme designed to gradually achieve saturation. We further lengthened the time between microdoses to elicit craving cycles, using both fixed-interval and subject-driven behavior. Finally, we demonstrated a potential application of our device by showing that a fixed-interval protocol can reliably identify neuromodulatory targets for pharmacotherapy or brain stimulation.

Our nasal drug delivery method consisted of a series of spaced microdoses rather than a one-time dose achieving immediate or consistent saturation. This has two significant methodological advantages. First, it makes it possible to neuroimage the dynamics of self-administration, since the satiety’s transience initiates further drug-seeking behavior. Second, the cycling reveals interactions between control sub-circuits necessary for computational modeling. Importantly, the delivery device and protocol can be adapted for use with other drugs that affect the brain (e.g., cocaine, opiates such as remifentanil with high potency and short half-life) by modulating dosage and inter-trial intervals, thereby providing a quantitative comparison of how neurobiological control dynamics, and their associated self-administrated behavior, differ across compounds. This will be particularly useful as clinical neuroimaging of addiction evolves beyond broad conceptual schemas, towards data-driven predictive models designed to rigorously quantify dysregulation and generate relapse trajectories at the single-subject level—a potential first step towards addiction-related personalized medicine.

Even though group-level studies are useful for discovering a generalizable set of brain regions responding to a psychostimulant, learning addiction dynamics need inference at an individual subject level [53]. Unfortunately, very little research has focused on single-subject-level analysis [54], which is an absolute necessity in clinical applications. Nicotine addiction affects neural subsystems associated with decision making, emotional processing, memory, motivation, salience, and interoception—the coupled effect of which introduces variable addiction dynamics for each subject. Our study focused on an MRI-compatible device development and reliably identifying neuromodulatory targets for pharmacotherapy or brain stimulation at a single-subject level. We elucidated a procedure to find neuromodulation targets non-invasively using our nicotine delivery device and fMRI, where the neuromodulation targets are driver nodes that control the regions connected to them, and thus can alter the underlying neuronal circuits at a synaptic level. While both pharmacological intervention via drugs like bupropion or varenicline [55], and brain stimulation techniques like transcranial magnetic stimulation (rTMS) [56,57,58], have shown promise as therapeutics in nicotine addiction, it is extremely difficult to probe neural pathways and neuromodulatory targets non-invasively for personalized treatment. Our device, along with recent analytical methods developed for single-subject-level analysis [59], can be used for probing oscillating antagonistic sub-circuits associated with addiction. These sub-circuits modulate repeated-cycle transitions between periods of craving (affecting the nucleus accumbens and prefrontal-limbic circuit associated with aversive stimuli/emotional stress [7,60,61]), reward following partial drug administration (affecting the nucleus accumbens and activating the substantia nigra subcomponents of the basal ganglia circuit [62]), and transient satiety (affecting the prefrontal-limbic circuit and the caudate and pallidum subcomponents of the basal ganglia circuit). Future efforts may be geared towards probing the antagonistic sub-circuits for single-subject fMRI with the current delivery device and establishing associated addiction dynamics for each individual. Beyond addiction research, our nasal delivery device can be employed using any nebulizable drug and could provide a promising path towards establishing nasal delivery pathways in humans by probing the dynamics of the brain’s neural response to nasal drug delivery. For example, there is evidence that direct transport of drugs from the nasal cavity into the brain depend on the drug’s lipophilicity [63,64]. Our method would allow to test such a hypothesis.

## Figures and Tables

**Table 1:** Characterization of Subjects.

Study		Gender		Age		Fagerstrom Score	
	N	M	F	Mean	SD	Mean	SD
*Study A*	17	3	14	33.64	11.63	6.58	0.69
*Study B*	15	3	12	33.13	10.99	6.75	0.86
*Study C*	8	2	6	39	9.94	7	1.19
*Study D*	4	2	2	37.5	6.85	8.25	0.5
*Study E*	5	2	3	34.6	6.42	7.4	1.34

**Table 2:** *Two most-dominant driver nodes for abstinence and satiety across all subjects.* The percentage-controllable nodes show the ratio of average number of nodes across all brain-connectivity states controlled by the driver node and the total number of brain regions (23). For subjects 4 and 5, the second node is not shown for the satiety condition as no other node than the ACC was a common driver across all brain-connectivity states.

Subject	Condition	Driver Nodes	Controllable Nodes (% of Total Nodes)
1	Abstinence	Nucleus Accumbens (Left)	42.39%
		Anterior Cingulate Cortex	36.95%
			
	Satiety	Anterior Cingulate Cortex	36.23%
		Nucleus Accumbens (Left)	28.98%
			
2	Abstinence	Anterior Cingulate Cortex	75.36%
		Amygdala (Left)	18.84%
			
	Satiety	Anterior Cingulate Cortex	58.69%
		Hippocampus (Left)	21.73%
			
3	Abstinence	Anterior Cingulate Cortex	49.28%
		Caudate (Left)	39.13%
			
	Satiety	Anterior Cingulate Cortex	62.31%
		Nucleus Accumbens (Left)	33.33%
			
4	Abstinence	Anterior Cingulate Cortex	62.31%
		Nucleus Accumbens (Left)	23.19%
			
	Satiety	Anterior Cingulate Cortex	69.56%

## Data Availability

The data presented in this study are available on request from the corresponding author.

## References

[1] Public Policy Statement on Nicotine Dependence and Tobacco. https://www.dshs.wa.gov/sites/default/files/BHSIA/dbh/documents/ASAM%20Position%20Paper%20on%20Nicotine%20Addiction.pdf.

[2] Benjamin R. (2010). How Tobacco Smoke Causes Disease: The Biology and Behavioral Basis for Smoking-Attributable Disease: A Report of the Surgeon General.

[3] Tobacco, Nicotine, and E-Cigarettes Research Report. https://www.drugabuse.gov/download/1344/tobacco-nicotine-e-cigarettes-research-report.pdf.

[4] D’Souza M.S., Markou A. (2013). The “Stop” and “Go” of Nicotine Dependence: Role of GABA and Glutamate. Cold Spring Harb. Perspect. Med..

[5] Markou A. (2008). Neurobiology of nicotine dependence. Philos. Trans. R. Soc. B Biol. Sci..

[6] Koob G.F., Volkow N.D. (2016). Neurobiology of addiction: A neurocircuitry analysis. Lancet Psychiatry.

[7] Mujica-Parodi L.R., Cha J., Gao J. (2017). From Anxious to Reckless: A Control Systems Approach Unifies Prefrontal-Limbic Regulation across the Spectrum of Threat Detection. Front. Syst. Neurosci..

[8] Mujica-Parodi L.R., Strey H.H. (2020). Making Sense of Computational Psychiatry. Int. J. Neuropsychopharmacol..

[9] Le Houezec J. (2003). Role of nicotine pharmacokinetics in nicotine addiction and nicotine replacement therapy: A review. Int. J. Tuberc. Lung Dis..

[10] DeDora D.J., Enedic S., Ekatti P., Earnab S., Wald L., Etakahashi A., Van Dijk K., Strey H., Mujica-Parodi L. (2016). Signal Fluctuation Sensitivity: An Improved Metric for Optimizing Detection of Resting-State fMRI Networks. Front. Neurosci..

[11] Kumar R., Tan L., Kriegstein A., Lithen A., Polimeni J.R., Mujica-Parodi L.R., Strey H.H. (2020). Ground-truth “resting-state” signal provides data-driven estimation and correction for scanner distortion of fMRI time-series dynamics. NeuroImage.

[12] Stead L.F., Perera R., Bullen C., Mant D., Hartmann-Boyce J., Cahill K., Lancaster T. (2012). Nicotine replacement therapy for smoking cessation. Cochrane Database Syst. Rev..

[13] Frederick B.D., Lindsey K.P., Nickerson L.D., Ryan E.T., Lukas S.E. (2007). An MR-compatible device for delivering smoked marijuana during functional imaging. Pharmacol. Biochem. Behav..

[14] Phipps P.R., Pharm B., Gonda I. (1990). Droplets Produced by Medical Nebulizers. Chest.

[15] Rose J., Rose J.E., Behm F.M., Westman E.C., Coleman R. (1999). Arterial nicotine kinetics during cigarette smoking and intravenous nicotine administration: Implications for addiction. Drug Alcohol Depend..

[16] Bourganis V., Kammona O., Alexopoulos A., Kiparissides C. (2018). Recent advances in carrier mediated nose-to-brain delivery of pharmaceutics. Eur. J. Pharm. Biopharm..

[17] Lochhead J., Thorne R.G. (2012). Intranasal delivery of biologics to the central nervous system. Adv. Drug Deliv. Rev..

[18] Illum L. (2003). Nasal drug delivery—possibilities, problems and solutions. J. Control. Release.

[19] Jones D.S., Craig D.Q.M. (2004). The Journal of Pharmacy and Pharmacology– four years on. J. Pharm. Pharmacol..

[20] Dhuria S.V., Hanson L.R., Frey W.H. (2010). Intranasal delivery to the central nervous system: Mechanisms and experimental considerations. J. Pharm. Sci..

[21] Erdő F., Bors L.A., Farkas D., Bajza Á., Gizurarson S. (2018). Evaluation of intranasal delivery route of drug administration for brain targeting. Brain Res. Bull..

[22] Fortuna A., Alves G., Serralheiro A., Sousa J., Falcão A. (2014). Intranasal delivery of systemic-acting drugs: Small-molecules and biomacromolecules. Eur. J. Pharm. Biopharm..

[23] Pires A., Fortuna A., Alves G., Falcão A. (2009). Intranasal Drug Delivery: How, Why and What for?. J. Pharm. Pharm. Sci..

[24] Crowe T., Greenlee M.H.W., Kanthasamy A., Hsu W.H. (2018). Mechanism of intranasal drug delivery directly to the brain. Life Sci..

[25] Schneider N.G., Lunell E., Olmstead R.E., Fagerström K.-O. (1996). Clinical Pharmacokinetics of Nasal Nicotine Delivery. Clin. Pharmacokinet..

[26] Cheng Y., Holmes T., Gao J., Guilmette R., Li S., Surakitbanharn Y., Rowlings C. (2001). Characterization of Nasal Spray Pumps and Deposition Pattern in a Replica of the Human Nasal Airway. J. Aerosol Med..

[27] Kashani A., Mostaghimi J. (2010). Aerosol characterization of concentric pneumatic nebulizer used in inductively coupled plasma—Mass spectrometry (icp-ms). At. Sprays.

[28] Heatherton T.F., Kozlowski L.T., Frecker R.C., Fagerstrom K.-O. (1991). The Fagerström Test for Nicotine Dependence: A revision of the Fagerstrom Tolerance Questionnaire. Br. J. Addict..

[29] Arora P., Sharma S., Garg S. (2002). Permeability issues in nasal drug delivery. Drug Discov. Today.

[30] Nitish Srivastava G.H., Alex K., Ilya S., Ruslan S. (2014). Dropout: A Simple Way to Prevent Neural Networks from Overfitting. J. Mach. Learn. Res..

[31] Falcato L., Beck T., Reimer J., Verthein U. (2015). Self-Reported Cravings for Heroin and Cocaine during Maintenance Treatment with Slow-Release Oral Morphine Compared with Methadone. J. Clin. Psychopharmacol..

[32] Giuliani N.R., Cosme D., Merchant J.S., Dirks B., Berkman E.T. (2020). Brain Activity Associated with Regulating Food Cravings Predicts Changes in Self-Reported Food Craving and Consumption Over Time. Front. Hum. Neurosci..

[33] Zello G.A., Smith J.M., Pencharz P.B., Ball R.O. (1990). Development of a Heating Device for Sampling Arterialized Venous Blood from a Hand Vein. Ann. Clin. Biochem. Int. J. Lab. Med..

[34] Ciuciu P., Varoquaux G., Abry P., Sadaghiani S., Kleinschmidt A. (2012). Scale-free and multifractal time dynamics of fMRI signals during rest and task. Front. Physiol..

[35] Smith S.M., Jenkinson M., Woolrich M.W., Beckmann C.F., Behrens T.E., Johansen-Berg H., Bannister P.R., De Luca M., Drobnjak I., Flitney D.E. (2004). Advances in functional and structural MR image analysis and implementation as FSL. NeuroImage.

[36] Najem B., Houssière A., Pathak A., Janssen C., Lemogoum D., Xhaët O., Cuylits N., van de Borne P. (2006). Acute Cardiovascular and Sympathetic Effects of Nicotine Replacement Therapy. Hypertension.

[37] Gordon E.M., Laumann T.O., Gilmore A.W., Newbold D.J., Greene D., Berg J.J., Ortega M., Hoyt C., Gratton C., Sun H. (2017). Precision Functional Mapping of Individual Human Brains. Neuron.

[38] Birn R.M. (2012). The role of physiological noise in resting-state functional connectivity. NeuroImage.

[39] Caballero-Gaudes C., Reynolds R.C. (2016). Methods for cleaning the BOLD fMRI signal. NeuroImage.

[40] Behzadi Y., Restom K., Liau J., Liu T.T. (2007). A component based noise correction method (CompCor) for BOLD and perfusion based fMRI. NeuroImage.

[41] Whitfield-Gabrieli S., Nieto-Castanon A. (2012). Conn: A Functional Connectivity Toolbox for Correlated and Anticorrelated Brain Networks. Brain Connect..

[42] Liu Y.-Y., Slotine J.E., Barabasi A. (2011). Controllability of complex networks. Nature.

[43] Gu S., Pasqualetti F., Cieslak M., Telesford Q.K., Yu A.B., Kahn A., Medaglia J.D., Vettel J.M., Miller M.B., Grafton S.T. (2015). Controllability of structural brain networks. Nat. Commun..

[44] Borsboom D. (2017). A network theory of mental disorders. World Psychiatry.

[45] Koob G.F., Volkow N.D. (2009). Neurocircuitry of Addiction. Neuropsychopharmacology.

[46] Virtanen P., Gommers R., Oliphant T.E., Haberland M., Reddy T., Cournapeau D., Burovski E., Peterson P., Weckesser W., Bright J. (2020). SciPy 1.0: Fundamental algorithms for scientific computing in Python. Nat. Methods.

[47] Nacher J.C., Akutsu T. (2016). Minimum dominating set-based methods for analyzing biological networks. Methods.

[48] Green J.H., Ellis F.R., Shallcross T.M., Bramley P.N. (1990). Invalidity of Hand Heating as a Method to Arterialize Venous Blood. Clin. Chem..

[49] Zhao Y., Sallie S.N., Cui H., Zeng N., Du J., Yuan T., Li D., De Ridder D., Zhang C. (2020). Anterior Cingulate Cortex in Addiction: New Insights for Neuromodulation. Neuromodulation Technol. Neural Interface.

[50] Bari A., DiCesare J., Babayan D., Runcie M., Sparks H., Wilson B. (2018). Neuromodulation for substance addiction in human subjects: A review. Neurosci. Biobehav. Rev..

[51] Lozano A.M., Lipsman N. (2013). Probing and Regulating Dysfunctional Circuits Using Deep Brain Stimulation. Neuron.

[52] Wang T.R., Moosa S., Dallapiazza R.F., Elias W.J., Lynch W.J. (2018). Deep brain stimulation for the treatment of drug addiction. Neurosurg. Focus.

[53] Van der Stel J. (2015). Precision in Addiction Care: Does It Make a Difference?. Yale J. Biol. Med..

[54] Fadiga L. (2007). Functional magnetic resonance imaging: Measuring versus estimating. NeuroImage.

[55] Jain R., Majumder P., Gupta T. (2013). Pharmacological Intervention of Nicotine Dependence. BioMed Res. Int..

[56] Wing V.C., Barr M.S., Wass C.E., Lipsman N., Lozano A.M., Daskalakis Z.J., George T.P. (2013). Brain Stimulation Methods to Treat Tobacco Addiction. Brain Stimul..

[57] Salling M.C., Martínez D. (2016). Brain Stimulation in Addiction. Neuropsychopharmacology.

[58] Tseng P., Jeng J., Zeng B., Stubbs B., Carvalho A.F., Brunoni A.R., Su K., Tu Y., Wu Y., Chen T. (2021). Efficacy of non-invasive brain stimulation interventions in reducing smoking frequency in patients with nicotine dependence: A systematic review and network meta-analysis of randomized controlled trials. Addiction.

[59] Kumar R., Strey H.H., Mujica-Parodi L.R. (2021). Quantifying control circuit regulation in the human brain. bioRxiv.

[60] LeDoux J. (2003). The emotional brain, fear, and the amygdala. Cell Mol. Neurobiol..

[61] Phelps E.A., LeDoux J.E. (2005). Contributions of the Amygdala to Emotion Processing: From Animal Models to Human Behavior. Neuron.

[62] Morita K., Morishima M., Sakai K., Kawaguchi Y. (2013). Dopaminergic Control of Motivation and Reinforcement Learning: A Closed-Circuit Account for Reward-Oriented Behavior. J. Neurosci..

[63] Inoue D., Furubayashi T., Tanaka A., Sakane T., Sugano K. (2020). Effect of Cerebrospinal Fluid Circulation on Nose-to-Brain Direct Delivery and Distribution of Caffeine in Rats. Mol. Pharm..

[64] Sakane T., Akizuki M., Yamashita S., Nadai T., Hashida M., Sezaki H. (1991). The Transport of a Drug to the Creebrospinal Fluid Directly from the Nasal Cavity: The Relation to the Lipophilicity of the Drug. Chem. Pharm. Bull..

